# Compositional and Functional Shifts in the Epibiotic Bacterial Community of *Shinkaia crosnieri* Baba & Williams (a Squat Lobster from Hydrothermal Vents) during Methane-Fed Rearing

**DOI:** 10.1264/jsme2.ME18072

**Published:** 2018-10-17

**Authors:** Tomo-o Watsuji, Kaori Motoki, Emi Hada, Yukiko Nagai, Yoshihiro Takaki, Asami Yamamoto, Kenji Ueda, Takashi Toyofuku, Hiroyuki Yamamoto, Ken Takai

**Affiliations:** 1 Japan Agency for Marine-Earth Science and Technology (JAMSTEC) 2–15 Natsushima-cho, Yokosuka, Kanagawa 237–0061 Japan; 2 Life Science Research Center, College of Bioresource Sciences, Nihon University 1866 Kameino, Fujisawa 252–0880 Japan

**Keywords:** energy and carbon sources, epibiotic bacterial community, cross-feeding, chemosynthetic ecosystem

## Abstract

The hydrothermal vent squat lobster *Shinkaia crosnieri* Baba & Williams harbors an epibiotic bacterial community, which is numerically and functionally dominated by methanotrophs affiliated with *Methylococcaceae* and thioautotrophs affiliated with *Sulfurovum* and *Thiotrichaceae*. In the present study, shifts in the phylogenetic composition and metabolic function of the epibiont community were investigated using *S. crosnieri* individuals, which were reared for one year in a tank fed with methane as the energy and carbon source. The results obtained indicated that indigenous predominant thioautotrophic populations, such as *Sulfurovum* and *Thiotrichaceae* members, became absent, possibly due to the lack of an energy source, and epibiotic communities were dominated by indigenous *Methylococcaceae* and betaproteobacterial methylotrophic members that adapted to the conditions present during rearing for 12 months with a supply of methane. Furthermore, the overall phylogenetic composition of the epibiotic community markedly changed from a composition dominated by chemolithotrophs to one enriched with cross-feeding heterotrophs in addition to methanotrophs and methylotrophs. Thus, the composition and function of the *S. crosnieri* epibiotic bacterial community were strongly affected by the balance between the energy and carbon sources supplied for chemosynthetic production as well as that between the production and consumption of organic compounds.

Some species of crustaceans and polychaete annelids that dwell in deep-sea hydrothermal vents and cold seep environments harbor bacteria (epibionts) that adhere to the surfaces of their specialized tissues (3, 6, 11, 26, 35, 40). These epibionts are considered to be nutrient sources for host animals. A recent study demonstrated that the deep-sea squat lobster *Shinkaia crosnieri*, which inhabits hydrothermal vent fields in the Okinawa Trough, utilizes epibiotic bacteria as a primary nutrient source (43). A dense epibiotic community develops on numerous setae along the ventral aspect of *S. crosnieri* (40), and the squat lobster ingests and digests the epibionts harvested by the third maxillipeds (43).

Phylogenetic analyses of the epibionts of crustaceans endemic to hydrothermal vents and cold seeps have shown that phylotypes belonging to the genus *Sulfurovum* within the class *Epsilonproteobacteria* and the family *Thiotrichaceae* within the class *Gammaproteobacteria* typically dominate epibiotic bacterial communities (11, 25, 35, 40, 46). Long and thick filaments have been observed as common morphological features of *Sulfurovum*-affiliated epibionts (11, 25, 40). A ^13^C-labeled tracer experiment using nano-scale secondary ion mass spectrometry and fluorescence *in situ* hybridization (NanoSIMS-FISH) demonstrated that the *Sulfurovum*-affiliated epibionts of *S. crosnieri* are thioautotrophs (41). In contrast, the morphological features of *Thiotrichaceae*-affiliated epibionts are known to be short and thin filaments (25). The *Thiotrichaceae*-affiliated epibionts of vent-endemic crustaceans are also potential thioautotrophs (27, 40, 45). Moreover, *S. crosnieri* has been suggested to host abundant amounts of methanotrophs (40). Based on the transcription analysis of a functional gene (*pmoA*) encoding a subunit of particulate methane monooxygenase (pMMO, EC 1.14.18.3), epibiotic methanotrophs were identified as members of the family *Methylococcaceae* within *Gammaproteobacteria* (42) and a FISH analysis targeting 16S rRNA also found that the oval cells of *Methylococcaceae* dominated the epibiotic community of *S. crosnieri* (40).

Abundant thioautotrophic and methanotrophic populations in the epibiotic bacterial community appear to be sustained via the supply of energy and carbon sources, such as reduced sulfur compounds, methane, and inorganic carbons, in their habitats from hydrothermal fluid discharges. Thus, if the supply balance of their energy and carbon sources is naturally or artificially changed during the lifetime of *S. crosnieri*, the compositional and functional balance of their epibiotic bacterial community may also respond to the supply balance changes. To test this hypothesis, we performed a rearing experiment in the present study. *S. crosnieri* from the Okinawa Trough deep-sea hydrothermal systems is the only organism that is known to sustain its nutrition using both methanotrophic and thioautotrophic epibionts (43). Thus, we reared *S. crosnieri* individuals in a tank fed with methane for one year and assessed compositional and functional shifts in the epibiotic bacterial community based on microscopic observations, culture-dependent and -independent techniques, and functional measurements.

## Materials and Methods

### Collection of *S. crosnieri* from a deep-sea hydrothermal field

*S. crosnieri* individuals were obtained from the Iheya North hydrothermal field in the Okinawa Trough, Japan, during dive #1612 on 20 January 2014 (27° 48.00′ N, 126° 53.81′ E, at a water depth of 1002 m) and dive #1618 on 29 January 2014 (27° 47.45′ N, 126° 53.81′ E, at a water depth of 988 m) using the JAMSTEC remotely operated vehicle (ROV) ‘*HyperDolphin*’. They were collected from deep-sea vent habitats using a suction sampler and stored in a confined box filled with chilled seawater in the ROV. The *S. crosnieri* individuals obtained from dive #1612 were used in ^13^C-tracer experiments and a stable carbon isotope analysis as controls before methane-fed rearing experiments. Individuals from dive #1618 were employed for methane-fed rearing and in the other experiments described below.

### Methane-fed rearing

*S. crosnieri* individuals were reared for one year in a tank (80×45×45 cm) with a lid containing 100 L of artificial seawater (REISEA MARINE; Iwaki Pumps, Japan), which was fed with methane under atmospheric pressure (Fig. S1). The seawater in the tank was filtered continuously using a canister filter (Eheim Professional 3 2075; Eheim, Germany). The temperature of the seawater was controlled at 4.4–5.1°C via a connection between a thermostat controller (TC-100; Iwaki Pumps) and cooler (AZ-251X; Iwaki Pumps). The concentration of dissolved oxygen (DO) in the seawater was controlled at 2.5±0.007 mg L^−1^ (79±0.2 μmol L^−1^, mean±SE) via a connection between a DO sensor (Liquisys M COM223/253; Endress+Hauser, Germany) and air pump to supply O_2_ consumed by *S. crosnieri* and microorganisms. The pH of the seawater was controlled at 7.6±0.004 (mean±SE) by laying coral sand on the bottom of the tank to serve as a buffering agent. The pH value was measured continuously during rearing using a pH sensor (MicropH; Aquabase, Japan). Methane gas was dissolved in the seawater as follows. Using a water pump (PSD-10A; Iwaki Pumps) placed inside the tank, seawater was pumped into a bundle of hollow fibers (20E0240A3; Mitsubishi Rayon, Japan) in a housing placed outside the tank and returned to the tank. The housing was filled with methane gas at 105 kPa from a 10-L cylinder and the methane gas filtered from outside the hollow fibers was dissolved in the seawater passing through the hollow fibers. The dissolved methane in seawater was kept at a final concentration of 34±3 μM (mean±SE) during rearing. A 5-mL aliquot of rearing seawater was sampled every week from the tank and the dissolved gas components were extracted in a 69-mL vacuum vial (V-50; Nichiden-Rika Glass, Japan) with a butyl rubber stopper. The extracted gas components were measured using a gas chromatograph (GC-4000; GL Science, Japan) with a pulsed discharge detector and column packed with Molecular Sieve 5A (GL Science), and dissolved methane concentrations were measured.

### ^13^C-tracer experiments and stable carbon isotope analysis

*S. crosnieri* individuals immediately after capture and after methane-fed rearing for 3 and 12 months were used in ^13^C-labeled tracer experiments in onboard and onshore laboratories. Live individuals were incubated in 1 L of artificial seawater containing of 1 mmol [^13^C]methane (99% ^13^C) and 1 mmol sodium [^13^C]bicarbonate (99% ^13^C) in the presence and absence of 200 μmol of sodium sulfide, as described previously (40), and were removed and stored at −80°C. In addition, *S. crosnieri* individuals before and after methane-added rearing for 3 and 12 months were stored at −80°C for the natural stable isotope abundance analysis. The setae of the individuals stored at −80°C were subsampled onshore and the ^13^C composition of samples was assessed using a mass spectrometer (Delta Plus XP; Thermo Finnigan, Bremen, Germany), which was coupled online via a Finnigan ConFlo III interface with an elemental analyzer (FlashEA 1112; Thermo-Quest, Milan, Italy). All of the samples were analyzed in triplicate.

### Measurements of methane and sulfide oxidation

The methane-oxidizing activities of three *S. crosnieri* individuals were examined after rearing for 3 and 12 months by a previously described continuous-flow incubation method (42). The carapace lengths of the three individuals after rearing for 3 and 12 months were 36, 38, and 39 mm and 35, 37, and 39 mm, respectively. Artificial seawater and dissolved methane were prepared as described previously (42). Artificial seawater used in the experiment contained dissolved methane at a final concentration of approximately 20 μM. Artificial seawater was supplied at a flow rate of 4 mL min^−1^ at 5°C for 70 min and the consumption of dissolved methane was measured.

The sulfide-oxidizing activities of three *S. crosnieri* individuals were examined after rearing for 3 and 12 months by a batch incubation method. The carapace lengths of the three individuals after rearing for 3 and 12 months were 31, 33, and 33 mm and 32, 33, and 33 mm, respectively. Each of the *S. crosnieri* individuals was incubated at 5°C in a 295-mL glass bottle that was sealed with a butyl rubber stopper and contained 200 mL of filtered artificial seawater. Artificial seawater contained 130 μM sodium sulfide and 1 mM sodium bicarbonate at final concentrations. Artificial seawater in the bottle was subsampled at 20-min intervals during a 60-min incubation. The concentration of sulfide in the samples was assessed using the methylene blue method (9).

### Nucleic acid extraction and bacterial 16S rRNA and *pmoA* gene clone analyses

DNA extracts were obtained from the setae of three *S. crosnieri* individuals before and after rearing for 3 and 12 months, as well as the epibiotic bacteria isolated from *S. crosnieri* setae after rearing for 12 months (described below). Total DNA extraction as well as the amplification and sequencing of the 16S rRNA and *pmoA* genes were conducted as described previously (42, 43). Clustering of the single-stranded read 16S rRNA gene sequences with lengths of approximately 0.7 kb defined with a >97% identity threshold were performed as described previously (43). A representative clone of each OTU was further sequenced and an approximately 1.4-kb fragment of the 16S rRNA gene was assessed from both strands. The taxonomy of the representative sequences was assigned using the Wang method (MOTHUR version 1.35.1) against the SILVA version 132 database. In addition, representative 16S rRNA gene sequences were aligned with MUSCLE version 3.8.31 (7) and trimmed using trimAl version 1.2 (2). Best-fitting substitution models were selected by AIC values in jModelTest 2 version 2.1.10 (4). A maximum likelihood tree was inferred using RAxML version 8.2.9 software (29) with the GTRGAMMAI model and rapid bootstrapping for 1,000 iterations. Clustering of the partial PmoA amino acid sequences deduced from the gene sequences (showing >95% amino acid sequence identity) and the identification of representative clones were performed as described previously (42). The alignment of representative amino acid sequences was achieved with MAFFT version 7.312 (18). The alignment was trimmed using trimAl version 1.2 (2). The mtZoa protein substitution model with auto-correlated discrete gamma distribution was selected by evaluating Akaike Information Criterion (AIC) values in Aminosan (31). A maximum-likelihood tree was inferred using RAxML 8.2.9 software (29) with the MTZOA+GAMMA model and rapid bootstrapping for 300 iterations.

### FISH analysis

The setae dissected from *S. crosnieri* individuals after rearing for 3 and 12 months were fixed overnight with 4% paraformaldehyde in phosphate-buffered saline at 4°C, and then stored in 50% ethanol with phosphate-buffered saline at −30°C prior to a FISH analysis. Hybridization with the MEG2 and EPI653 probes was performed as described previously (40). The MEG2 and EPI653 probes were designed to detect epibionts affiliated with *Methylococcaceae* and *Sulfurovum*, respectively (40). The MEG2 and EPI653 probes were labeled with Cy3 and Alexa 488, respectively, as described previously (40).

### Electron microscopic observations

After rearing for 12 months, the setae of a *S. crosnieri* individual were used for transmission electron microscopy (TEM) and field emission-scanning electron microscopy (SEM) observations. Setae were fixed overnight with 2.5% (v/v) glutaraldehyde in artificial seawater at 4°C and then washed in filtered artificial seawater. Samples were prepared and observed as described previously (40).

### Isolation of epibiotic bacterial strains

After rearing for 12 months, the setae from an *S. crosnieri* individual were washed and homogenized in filtered artificial seawater. Serial dilutions were spread on plates containing marine 2216 agar (BD, USA) and METO medium with agar for methylotrophs (described below). The plates were then incubated at 20°C under an air atmosphere for two weeks. Representative colonies with different appearances were inoculated onto new plates and pure cultures were obtained on agar plates at 20°C under an air atmosphere. The agar plates with METO medium comprised (L^−1^ of distilled, deionized water): 25.0 g NaCl, 0.14 g K_2_HPO_4_, 0.14 g CaCl_2_, 3.4 g MgSO_4_·7H_2_O, 4.2 g MgCl_2_·6H_2_O, 0.33 g KCl, 0.25 g NaNO_3_, 0.5 mg NiCl_2_·6H_2_O, 0.5 mg Na_2_SeO_3_·5H_2_O, 1.1 mg Na_2_WO_4_, 10 mg Fe(NH_4_)_2_(SO_4_)_2_·6H_2_O, and 0.25 mg CuSO_4_·5H_2_O, and 10 mL trace mineral solution (1), 10 mL vitamin solution (1), 5.0 mL CH_3_OH, and 12.0 g agar. Final pH was adjusted to 7.6. Purity was confirmed by a microscopic examination and by the repeated partial sequencing of the 16S rRNA gene using the oligonucleotide primers Bac27F and Uni1492R (21).

### Characterization of isolated epibiotic bacteria

The growth characteristics of epibiotic bacteria isolated from the setae of an *S. crosnieri* individual after 12 months of rearing were tested for methanotrophy in MJmet medium (15), for thioautotrophy in MMJS medium (44), for methylotrophy in METO medium (agar removed from the medium described above), and for heterotrophy in marine broth 2216 (MB) medium (BD, USA). MJmet and MMJS media were prepared with headspace gas phases containing CH_4_, N_2_, CO_2_, and O_2_ (49:40:10:1; 200 kPa), and N_2_, CO_2_, and O_2_ (94:5:1; 200 kPa), respectively. The final pH value of each medium was adjusted to 7.6. METO and MB media were used under air. All media were incubated at 20°C. The growth of epibiotic bacteria in liquid media was measured by direct cell counting after staining with 4′,6-diamidino-2-phenylindole (28) using a phase-contrast microscope (BX53; Olympus, Japan).

## Results

### ^13^C-tracer experiments and a stable carbon isotope analysis

The assimilation of ^13^C-labeled carbon by epibiotic populations was examined using live *S. crosnieri* individuals immediately after onboard recovery and after methane-fed rearing for 3 and 12 months (Table 1). Compared with natural abundance δ^13^C values in epibiotic populations before and after rearing, [^13^C]methane was assimilated by all epibiotic populations before and after rearing. A greater abundance of [^13^C]methane was assimilated by epibiotic populations during rearing, particularly in longer periods of rearing, than before rearing. The assimilation of [^13^C]bicarbonate by epibiotic populations was detected before rearing and increased in the presence of sulfide added as a potential energy source for thioautotrophs (561 to 3530‰). The assimilation of [^13^C] bicarbonate by epibiotic populations after rearing for 3 months was not detected, even in the presence of sulfide (−40.1 to −31.7‰), while after rearing for 12 months, epibiotic populations were able to incorporate [^13^C]bicarbonate, whereas abundances were markedly lower than those in populations before rearing and were not significantly enhanced in the presence of sulfide (94.3 to 76.8‰) (unpaired *t*-test, *P*>0.05).

### Measurement of methane and sulfide oxidation

After rearing for 3 and 12 months, we measured methane-oxidizing activity in live *S. crosnieri* individuals using a continuous-flow apparatus. Dissolved methane concentrations were lower in effluent seawater than in influent seawater, even in negative control experiments without an *S. crosnieri* individual (Table 2). This may have been due to the diffusion of methane gas through the silicone tube and the formation of methane gas bubbles in the flow line path (42). The methane consumption rates of *S. crosnieri* individuals after rearing were higher than those of the negative controls, and the methane-oxidizing activity of *S. crosnieri* (its epibiotic bacterial community) was maintained throughout rearing. However, after rearing for 3 and 12 months, the average net rates of methane oxidation in *S. crosnieri* individuals decreased by 37 and 53%, respectively, from that before rearing (Table 2).

After rearing for 3 and 12 months, we also measured sulfide-oxidizing activity in live *S. crosnieri* individuals using the continuous-flow apparatus. However, this activity was low and the continuous-flow method did not successfully estimate activities in individuals after rearing (data not shown). Thus, sulfide-oxidizing activity rates in live *S. crosnieri* individuals after rearing were estimated based on the time course of sulfide consumption in batch incubations. In these experiments, sulfide concentrations decreased in the absence of an *S. crosnieri* individual (Table 3) due to chemical oxidation by O_2_ (41). The net sulfide consumption rates in *S. crosnieri* individuals after rearing were very low, and the net average rates in *S. crosnieri* individuals (their epibiotic bacterial communities) after rearing for 3 and 12 months decreased by 98 and 97%, respectively, from that estimated before rearing using the continuous-flow method (Table 3).

### Comparison of the epibiotic phylotype composition before and after rearing

A total of 98, 116, and 128 bacterial 16S rRNA gene clones were sequenced in clone libraries from the epibionts of *S. crosnieri* individuals before and after rearing for 3 and 12 months, respectively. Clone sequences that shared >97% identity were classified into the same phylotype. Most (99% abundance) of the phylotypes before rearing were members of *Sulfurovum* within *Epsilonproteobacteria* or *Thiotrichaceae* and *Methylococcaceae* within *Gammaproteobacteria* (Table S1 and Fig. S2). None of the *Sulfurovum*-affiliated phylotypes were obtained from clone libraries based on the epibiotic communities of *S. crosnieri* individuals after rearing for 3 and 12 months (Table S1 and Fig. S2). In contrast, sequences affiliated with *Thiotrichaceae* were detected in epibiont clone libraries after rearing and were assembled into one phylotype (12 methane_07) with a close relationship to *Cocleimonas flava* (97% identity) (Table S1 and Fig. S2), which is a sulfur-oxidizing and heterotrophic bacterium isolated from a sand snail (32). Sequences affiliated with *Methylococcaceae* were also detected in epibiont clone libraries after rearing, and one of the main phylotypes in the library before rearing (12 methane_1-14) remained as an abundant phylotype in epibiont clone libraries after rearing (Table S1 and Fig. S2). In addition to phylotypes affiliated with the main phylogenetic groups in the indigenous epibiotic bacterial community, phylotypes belonging to *Alphaproteobacteria*, *Betaproteobacteria*, *Deltaproteobacteria*, and *Bacteroidetes* as well as others were detected in epibiont clone libraries from *S. crosnieri* individuals after rearing (Table S1 and Fig. S2). The most common phylotype (12 methane_01) representing 25 and 23% of epibiotic clone libraries after rearing for 3 and 12 months, respectively, was classified into the family *Methylophilaceae* within *Betaproteobacteria* (Table S1 and Fig. S2) and was related to *Methylotenera versatilis* (95% identity), which may grow with methanol as the sole carbon and energy source (17).

### Phylogenetic analysis using deduced PmoA amino acid sequences

**Fig. 1:**
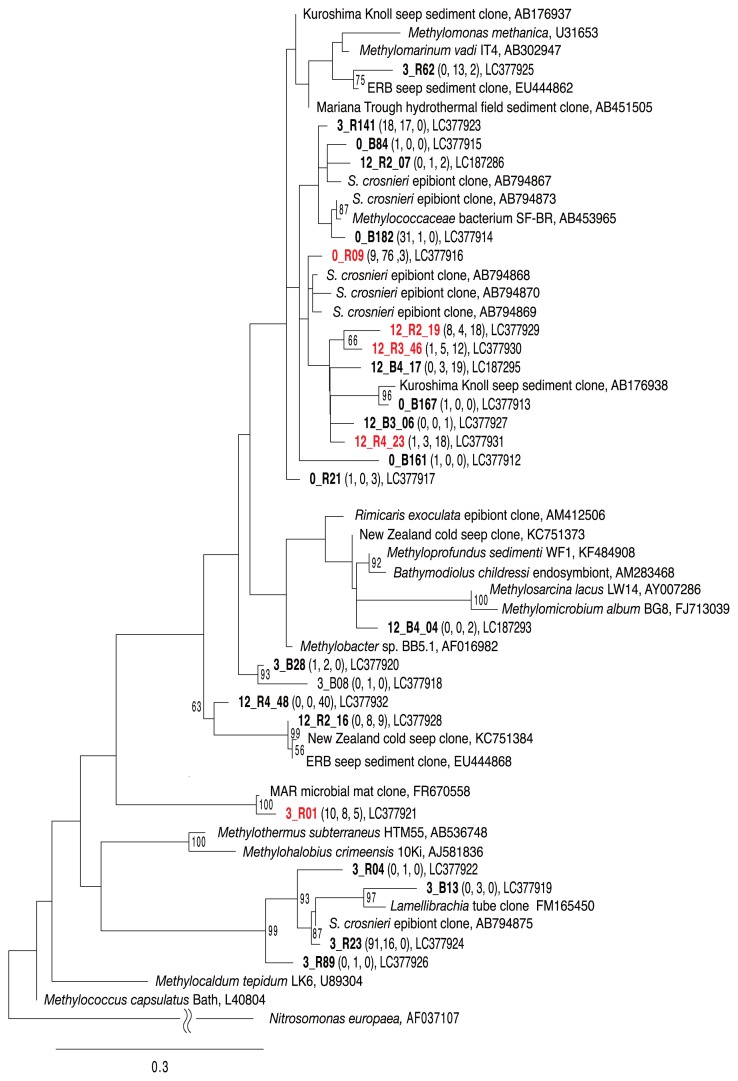
Phylogenetic tree based on partial amino acid sequences of the pMMO subunit A, deduced from *pmoA* sequences. Ammonia monooxygenase 1 subunit A, deduced from the *amoA* sequences of *Nitrosomonas europaea*, was defined as an outgroup. The deduced sequences of 157 amino acid residues were analyzed by maximum likelihood. Sequences obtained from the *S. crosnieri* epibiotic community in this study are shown in bold. The initial letters (B and R) of the sequence names indicate the *pmoA* gene sequence amplified with the primers A189f/mb661r and primers A189f/A682r, respectively. Numbers in parentheses indicate the numbers of clones retrieved from the *S. crosnieri* epibiotic community before and after rearing for 3 and 12 months, respectively. Sequences consistently obtained from the epibiotic community before and after rearing are shown in red. A bootstrap analysis was performed with 300 resampled data sets. Bootstrap values >50% are shown at branch points. The scale bar indicates 0.3 substitutions per site.

A total of 173, 163, and 132 *pmoA* sequences encoding a subunit of pMMO that is essential for aerobic methane-oxidizing metabolism were retrieved from the DNA extracts of epibiotic populations from *S. crosnieri* individuals before and after rearing for 3 and 12 months, respectively. Twenty-three representative *pmoA*-derived amino acid sequences (with an identity threshold >95%) were identified based on comparisons of partial PmoA amino acid sequences deduced from DNA sequences. The representative sequences were subjected to a phylogenetic analysis because the phylogenetic relationships of deduced PmoA amino acid sequences may be congruent with phylogeny based on 16S rRNA gene sequences (13, 19). All the representative *pmoA*-derived amino acid sequences were classified into *Methylococcaceae* and were related to those deduced from the cDNA sequences of the *S. crosnieri* epibiotic community as well as the DNA sequences from cold seeps and hydrothermal vent fields (Fig. 1). In addition, five representative *pmoA*-derived amino acid sequences were consistently detected in the epibiotic community before and after rearing (Fig. 1).

### FISH analysis

**Fig. 2:**
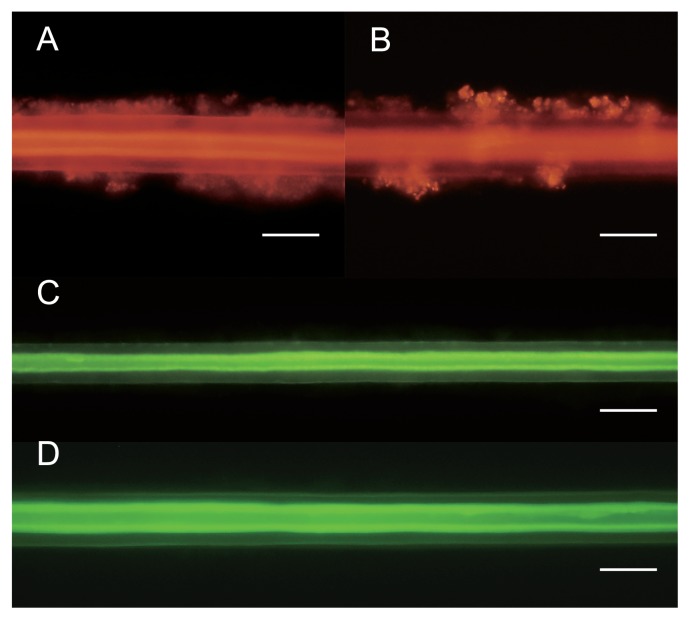
Fluorescence microscopy of setae of *S. crosnieri* individuals during methane-fed rearing. Fluorescence microscopy was performed for the setae of *S. crosnieri* after rearing for 3 months (A and C) and 12 months (B and D). FISH with the MEG2 probe specifically detects *Methylococcaceae*-affiliated epibionts on the setae (A and B). FISH with the EPI653 probe specifically detects *Sulfurovum*-affiliated epibionts on setae (C and D). Scale bars indicate 20 μm (A and B) and 30 μm (C and D).

A FISH analysis targeting the 16S rRNA sequences of epibionts affiliated with *Methylococcaceae* and *Sulfurovum* was performed using the setae of *S. crosnieri* after rearing for 3 and 12 months. Intrinsic fluorescence by *S. crosnieri* setae was observed in all specimens, as described previously (43). Epibionts related to *Methylococcaceae* members were detected specifically based on the fluorescence of the Cy3-labeled probe in epibiotic communities after rearing for 3 and 12 months (Fig. 2A and B). After rearing for 12 months, *Methylococcaceae*-affiliated epibionts were oval in shape (Fig. 2B). In contrast, *Sulfurovum*-affiliated epibionts with long and thick filaments (41) were not detected based on the fluorescence of the Alexa 488-labeled probe in epibiotic communities after rearing (Fig. 2C and D).

### Microscopic observations

An SEM analysis showed that after rearing for 12 months, setae from the walking leg of an *S. crosnieri* individual remained covered with microbial populations, and filamentous microbes were occasionally observed on the setae (Fig. S3A and B). High-magnification SEM images showed that the epibiotic bacterial community was dominated by morphotypes comprising short- and thin-filament cells and oval cells (Fig. S3B). TEM images also showed that oval cells on the setae contained intracytoplasmic membranes, which are a typical morphological feature of methanotrophs within *Methylococcaceae* (14, 16) (Fig. S3C). Large low electron density inclusions, presumably containing polyhydroxyalkanoate, were also observed in the oval cells (Fig. S3C).

### Characterization of isolated epibiotic bacteria

After rearing for 12 months, the heterotrophic strains KP105, DN153, and KT915 were isolated using MB medium from the epibiotic bacterial community, and the heterotrophic strain KM513 was isolated using METO medium (Table 4). The nearly complete 16S rRNA gene sequences of these strains were closely related to phylotypes obtained from epibiotic bacterial communities after rearing for 3 and 12 months, such as 12 methane_07 (belonging to *Thiotrichaceae*) for strain KP105, 12 methane_28 (belonging to *Rhodobacteraceae*) for strain DN153, 3 methane_1-13 (belonging to *Flavobacteriaceae*) for strain KT915, and 12 methane_17 (belonging to *Methylophilaceae*) for strain KM513 with 97, 99, 97, and 98% identities, respectively (Table S1 and Fig. S2). The *pmoA* gene was not amplified in any of the DNA extracts obtained from the isolated strains (Table 4). Only strain KM513 belonging to *Methylophilaceae* was capable of growth on METO medium with methanol as the sole energy and carbon source (Table 4). None of the strains grew in MMJS and MJmet media for thioautotrophs and methanotrophs, respectively (Table 4).

## Discussion

As shown in previous studies (40, 41, 43), a 16S rRNA gene clone analysis of epibiotic communities before rearing showed that they primarily comprised typical bacterial thioautotrophic and methanotrophic phylotypes belonging to the genus *Sulfurovum* and the families *Thiotrichaceae* and *Methylococcaceae* (Table S1 and Fig. S2). However, after rearing for 3 and 12 months in a methane-fed tank, 16S rRNA gene clone and microscopic analyses showed that *Sulfurovum*-affiliated populations disappeared from epibiotic communities (Table S1 and Fig. 2). The incorporation of [^13^C]bicarbonate in the epibiotic communities of *S. crosnieri* individuals was markedly less after rearing for 3 and 12 months than before rearing, and was not enhanced by the presence of sulfide (Table 1). These results indicate that *S. crosnieri* individuals lost their *Sulfurovum*-affiliated populations and their thioautotrophic function during methane-fed rearing for 12 months and even for 3 months.

The abundances and functions of *Thiotrichaceae*-affiliated thioautotrophic epibionts as well as *Sulfurovum* decreased during methane-fed rearing. We confirmed that the *S. crosnieri* epibiotic community assimilated inorganic carbon without the addition of an energy source before rearing (Table 1). A previous study suggested that *Thiotrichaceae* epibionts potentially assimilate inorganic carbon using sulfur stored within their cells and without an external supply of reduced sulfur compounds (41). However, inorganic carbon was not assimilated by *S. crosnieri* epibionts after rearing for 3 months, even in the presence of sulfide utilized by thioautotrophs as a potential energy source for chemosynthesis (Table 1). These results indicate that the active thioautotrophic function from the original epibiotic community was strongly affected by methane-fed rearing for 3 months. The 16S rRNA gene clone analysis also showed that the abundances of most of the *Thiotrichaceae* phylotypes in the epibiotic community were lost during rearing, whereas the clonal abundance of one phylotype (12 methane_07) of *Thiotrichaceae* increased during rearing (Table S1 and Fig. S2). The heterotrophic strain KP105 isolated from the epibiotic community of an *S. crosnieri* individual after rearing for 12 months was closely related to the phylotype 12 methane_07 (97% identity). The cultivation test indicated that this strain did not grow with methane (methanotrophy) and methanol (methylotrophy), or even with reduced sulfur compounds and inorganic carbons (thioautotrophy) (Table 4). The 97% 16S rRNA gene sequence identity between the heterotrophic strain KP105 and the phylotype 12 methane_07 does not necessarily represent similarities in their metabolic and physiological functions; however, the *Thiotrichaceae* populations detected in epibiotic communities during methane-fed rearing for 12 months appeared to function as heterotrophic consumers rather than as thioautotrophic primary producers.

In addition, the weak but detectable incorporation of [^13^C] bicarbonate independently of sulfide and slight sulfide consumption were observed in epibiotic communities after methane-fed rearing for 12 months (Tables 1 and 3). *Rhodobacteraceae*-affiliated phylotypes were detected in the 16S rRNA gene clone libraries of epibionts obtained from *S. crosnieri* individuals after rearing for 12 months (Table S1 and Fig. S2). Several members of *Rhodobacteraceae* are known to exhibit mixotrophic carbon metabolism (30, 33, 39). Thus, the potentially anaplerotic inorganic carbon fixation of potentially mixotrophic *Rhodobacteraceae*-affiliated and other heterotrophic epibionts may have contributed to the incorporation of [^13^C]bicarbonate independently of sulfide in the epibiotic communities after methane-fed rearing for 12 months. In addition, hydrogen sulfide (sulfide) is known to be toxic to animals because it may bind to cellular iron and disrupt the functions of mitochondria (8). The brachyuran crab, *Bythograea thermydron* Williams, which is endemic to deep-sea hydrothermal environments, may detoxify hydrogen sulfide (sulfide) via its oxidation to thiosulfate and sulfate using its own detoxification enzymes (37). Although we lack any physiological and genetic evidence, slight sulfide consumption by *S. crosnieri* individuals during methane-fed rearing may have been catalyzed by the function of the host *S. crosnieri* rather than by its epibiotic bacterial community.

[^13^C]methane assimilation and methane consumption experiments clearly indicated that *S. crosnieri* individuals reared in the methane-fed tank harbored active methanotrophs in their epibiotic communities even after 12 months (Tables 1 and 2). Previous studies demonstrated that the oval cells of *Methylococcaceae* members represented methanotrophic populations in the epibiotic communities of naturally living *S. crosnieri* individuals (40, 42). The 16S rRNA gene clone analysis demonstrated that the phylotypes affiliated with *Methylococcaceae* were preserved in the *S. crosnieri* epibiotic communities during methane-fed rearing for 12 months and one *Methylococcaceae* phylotype (12 methane_1-14) remained abundant throughout the rearing period (Table S1 and Fig. S2). The phylogenetic analysis of *pmoA* gene sequences showed that all of the potential methanotrophic populations present in *S. crosnieri* epibiotic communities throughout the rearing period were related to members of *Methylococcaceae* (Fig. 1). pMMO is present in almost all known methanotrophs, except for the genera *Methylocella* and *Methyloferula*, which are acidophilic methanotrophs within *Alphaproteobacteria* (5, 38), and the primer set used in the present study covered most of the known *pmoA* diversity (22). In addition, our microscopic observations using FISH and TEM analyses verified the abundant occurrence of *Methylococcaceae*-like oval cells in *S. crosnieri* epibionts even after rearing for 12 months (Fig. 2 and S3). Overall, these results strongly suggest that active *Methylococcaceae* methanotrophs were maintained as one of the predominant populations in *S. crosnieri* epibiotic communities when reared in the methane-fed tank for 12 months.

The net methane consumption rates by *S. crosnieri* individuals after rearing for 3 and 12 months decreased to approximately half of those before rearing (Table 2), whereas the rate of [^13^C]methane incorporation by the epibiotic community was greater after than before rearing (Table 1). These results may be explained by the effects of decreases in the biomass abundance and increases in the relative biomass proportion of methanotrophs in epibiotic communities during methane-fed rearing for 12 months. Although the setae of *S. crosnieri* immediately after capture are covered with more than 100 μm of filamentous microbes and have an abundant microbial biomass (36, 40), these very long filaments were not observed on the setae after rearing for 12 months (Fig. S3). In addition, typical long and thick filamentous *Sulfurovum*-affiliated epibionts were not observed after rearing for 3 and 12 months (Fig. 2). Thus, the overall biomass of the epibiotic community, including methanotrophic populations, continued to decrease whereas the relative abundances of the methanotrophic populations and their activities in the epibiotic communities increased with the methane-fed rearing period.

Stable isotope probing (SIP) with [^13^C]methane has been employed to investigate active methanotrophs in a number of environments (23, 24). However, the use of SIP techniques with a relatively long incubation time may lead to cross-feeding, through which non-targeted microorganisms incorporate the labeled substrates via secondary carbon flow from the targeted microorganisms (12). A methane-SIP study of sediments from an Arctic lake with active methane seepage indicated that [^13^C]methane incorporation was observed in members of various bacterial taxa, such as *Proteobacteria* (including *Methylococcaceae* and *Methylophilaceae*), *Bacteroidetes*, *Acidobacteria*, *Planctomycetes*, *Verrucomicrobia*, and *Actinobacteria* (12). After rearing for 3 and 12 months, the predominant phylotype components of the *S. crosnieri* epibiotic bacterial communities were similar to those found in the aforementioned [^13^C]methane-incorporating microbial community in Arctic lake sediments (Table S1 and Fig. S2). In addition, the bacterial strains isolated from the epibiotic community after rearing for 12 months, which were closely related to the phylotypes found in the 16S rRNA gene clone library of epibionts after rearing, were not methanotrophic, but instead were methylotrophic and heterotrophic (Table 4). Therefore, we conclude that the epibiotic communities of *S. crosnieri* individuals reared in the methane-fed tank changed from the original state dominated by chemolithotrophs (mainly thioautotrophic and methanotrophic populations) to a rearing-adapted state that mainly comprised residual methanotrophs and heterotrophs, which grow by cross-feeding on methanotrophically produced organic carbon.

Differences in hydrothermal fluid chemistry may have an effect on the epibiotic community composition of hydrothermal vent animals because epibiotic community compositions differed among the same species of animals from geographically distant and geologically different fields (23, 44). However, the relationship between the epibiotic community composition and the chemical environment of host animals’ habitats has not been clearly justified due to the difficulties associated with elucidating the chemical conditions of the habitat in detail. In the present study, *S. crosnieri* individuals were reared in a tank, the chemical environment of which was different from the natural habitat and was artificially controlled. The most prominent difference in the chemical environment was the availability of reduced sulfur compounds under *in situ* and methane-fed rearing conditions. In a previous study, the laboratory rearing of several *S. crosnieri* individuals was conducted in a tank fed with certain amounts of H_2_S and CO_2_ as the energy and carbon sources for 3 months (20). A preliminary 16S rRNA gene clone analysis of the epibiotic community suggested that after rearing for 3 months, *S. crosnieri* individuals hosted the *Sulfurovum* and *Thiotrichaceae* phylotypes, but lost the *Methylococcaceae* phylotypes (20). This preliminary rearing experiment did not fully explain the adaptive compositional and functional shifts in the epibiotic community of *S. crosnieri* during sulfide and CO_2_-fed rearing, but highlighted the effective impact of the energy source supply on the composition and function of the epibiotic community in *S. crosnieri.* The present study showed that the methane supply during rearing was a powerful environmental factor that induced compositional and functional shifts in the epibiotic community of *S. crosnieri*, which strongly suggests that the development of the *S. crosnieri* epibiotic community was affected by the balance between the energy and carbon sources supplied for primary production, and even by the balance between the production and consumption of organic compounds.

Previous studies provided molecular insights into the biological interactions between deep-sea vent-endemic chemosynthetic animals and their epibionts, in which *S. crosnieri* and the hydrothermal worm *Alvinella pompejana* Desbruyères & Laubier use an antimicrobial non-lectin polysaccharide and peptide to select particular bacterial members in the hydrothermal vent ecosystem (10, 34). The interaction between the functions of these environmental and biological factors may act as a selective force to control the compositional and functional development of epibiotic bacterial communities in the natural habitats of the deep-sea vent-endemic *S. crosnieri*, as well as other crustaceans and polychaete annelids. Rearing systems for deep-sea vent-endemic animals with artificial control over physical and chemical conditions in the habitat are effective for investigating the relationships between epibiotic microbial communities, host animals, and environmental conditions. *S. crosnieri* from the Okinawa Trough deep-sea hydrothermal systems may be reared for a relatively long time (>1 year) and may be useful as a model organism for future rearing-based chemosynthetic symbiosis research.

## Supplementary Material



## Figures and Tables

**Table 1:** Stable carbon isotope compositions of setae of a *S. crosnieri* individual during methane-fed rearing before and after tracer experiments

Rearing period (month)	δ^13^C (‰) of natural abundance	δ^13^C (‰) after labeled tracer experiments		

		[^13^C]methane	[^13^C]bicarbonate	[^13^C]bicarbonate +H_2_S
0	−40.3 0.2	278±30	561±8	3530±78

3	−38.8±1.0	612±41	—*	−36.0±0.9
	−40.0±0.4	676±19	—	−27.8±2.0
	−41.5±0.4	424±1	—	−31.4±1.0

(Average)	−40.1	571	—	−31.7

12	−41.4±0.4	1663±30	87.9±4.1	98.3±5.1
	−34.4±0.8	1052±40	44.8±6.6	20.9±5.8
	−41.2±0.2	1675±153	150±4	111±15

(Average)	−39.0	1463	94.3	76.8

Values were measured in triplicate and were expressed as means±standard deviations.

* No data were available

**Table 2:** Net methane consumption rates of live *S. crosnieri* individuals after methane-fed rearing

Rearing period (months)	Methane consumption rate (μmol h^−1^)		Net methane consumption rate (μmol h^−1^ individual^−1^)	Carapace length (mm)	Reference

	With an individual^a^	Without an individual^a^			
0	3.12±0.10	1.01	2.11±0.10	41	Watsuji *et al.* 2014
	2.46±0.13	1.01	1.45±0.13	37	Watsuji *et al.* 2014
	2.64±0.36	1.01	1.63±0.36	38	Watsuji *et al.* 2014

Average	2.74		1.73	39	

3	1.76±0.02	0.84	0.92±0.02	38	This study
	1.90±0.02	0.84	1.06±0.02	36	This study
	2.13±0.29	0.84	1.29±0.29	39	This study

Average	1.93		1.09	38	

12	1.73±0.09	1.01	0.72±0.09	39	This study
	1.83±0.08	1.01	0.82±0.08	37	This study
	1.91±0.14	1.01	0.90±0.14	35	This study

Average	1.82		0.81	37	

a All methane consumption rates were measured at 5-min intervals during the incubation and assessed using steady-state data.

They are expressed as means±s.d.

**Table 3:** Net sulfide consumption rates of live *S. crosnieri* individuals after methane-fed rearing

Rearing period (months)	Sulfide consumption rate (μmol h^−1^)		Net sulfide consumption rate (μmol h^−1^ individual^−1^)	Carapace length (mm)	Reference

	With an individual	Without an individual			
0	51.7	12.7	39.0	37	Watsuji *et al.* 2012

3	4.2^*^ (0.97)†	3.8^*^ (0.94)†	0.4	31	This study
	5.7 (0.85)	3.8 (0.94)	1.7	33	This study
	4.3 (0.89)	3.8 (0.94)	0.5	33	This study

(Average)	4.7		0.9	32	

12	2.4 (0.44)	3.8 (0.87)	0	32	This study
	5.7 (0.83)	3.8 (0.87)	1.9	33	This study
	4.9 (0.89)	3.8 (0.87)	1.2	33	This study

(Average)	4.3		1.1	33	

† The square of the correlation coefficient (R^2^) of each straight line was calculated.

**Table 4:** Representative isolates obtained from an epibiotic bacterial community of *S. crosnieri* individuals after 12 months of rearing

Isolate (Phylogenetic affiliation)	Detection of *pmoA*	Growth on each medium				Accession No.

		MMJS	MJmet	METO	MB	
(*Thiotrichaceae*) KP105	−	−	−	−	+	LC193132
(*Rhodobacteraceae*) DN153	−	−	−	−	+	LC193134
(*Flavobacteriaceae*) KT915	−	−	−	−	+	LC193136
(*Methylophilaceae*) KM513	−	−	−	+	w	LC193139

+, positive; −, negative; w, weakly positive
