# Supplementary material for: Compositional and Functional Shifts in the Epibiotic Bacterial Community of Shinkaia crosnieri Baba & Williams (a Squat Lobster from Hydrothermal Vents) during Methane-Fed Rearing
Source: Microbes Environ. 2018 Oct 17;33(4):348–56. doi: 10.1264/jsme2.ME18072 (PMC6308002; doi:10.1264/jsme2.ME18072)
Supplement: Supplementary file 1 [file 33_348_s1.pdf]

Fig. S1. Illustration of the methane-fed rearing system. The concentration of dissolved oxygen in seawater was controlled by a dissolved oxygen electrode (A), dissolved oxygen meter (B), and air pump (C). The concentration of dissolved methane in seawater was controlled by a methane gas cylinder (D), pressure regulator (E), water pump (F), pre-filter (G), and hollow fiber module (H). White arrows indicate the direction of gas flow. Black arrows indicate the direction of water flow.

Fig. S2. Phylogenetic tree based on epibiotic 16S rRNA gene sequences. The sequence of the 16S rRNA gene from *Aquifex aeolicus* was defined as an outgroup. Representative sequences obtained from the *S. crosnieri* epibiotic community in this study are shown in blue. Sequences of the bacterial strains isolated from the *S. crosnieri* epibiotic community after rearing for 12 months are shown in red. A bootstrap analysis was performed with 1,000 resampled data sets. Bootstrap values >50% are shown at branch points. The scale bar indicates 0.09 substitutions per site.

Fig. S3. Electron micrographs of setae of *S. crosnieri* individuals during methane-fed rearing. Scanning electron micrographs (A and B) and a transmission electron micrograph (C) of setae dissected from *S. crosnieri* after rearing for 12 months are shown. Scale bars indicate 20 μm (A), 5 μm (B), and 200 nm (C).

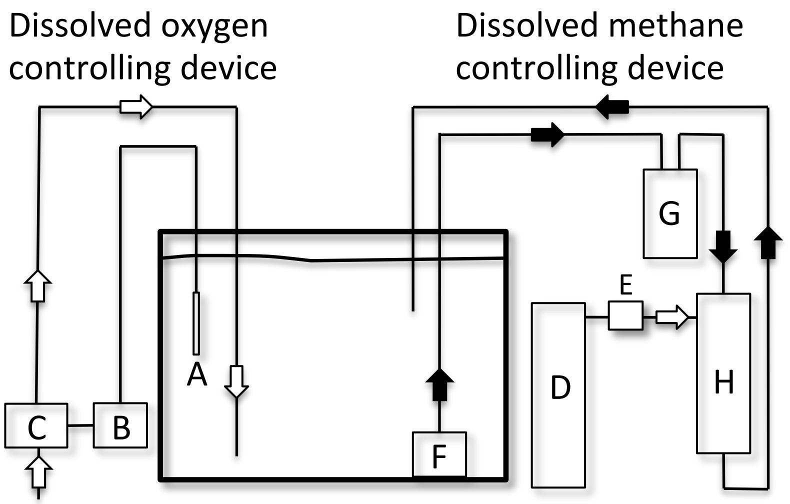
Dissolved oxygen
controlling device
Dissolved methane
controlling device
A
B
C
D
E
F
G
H

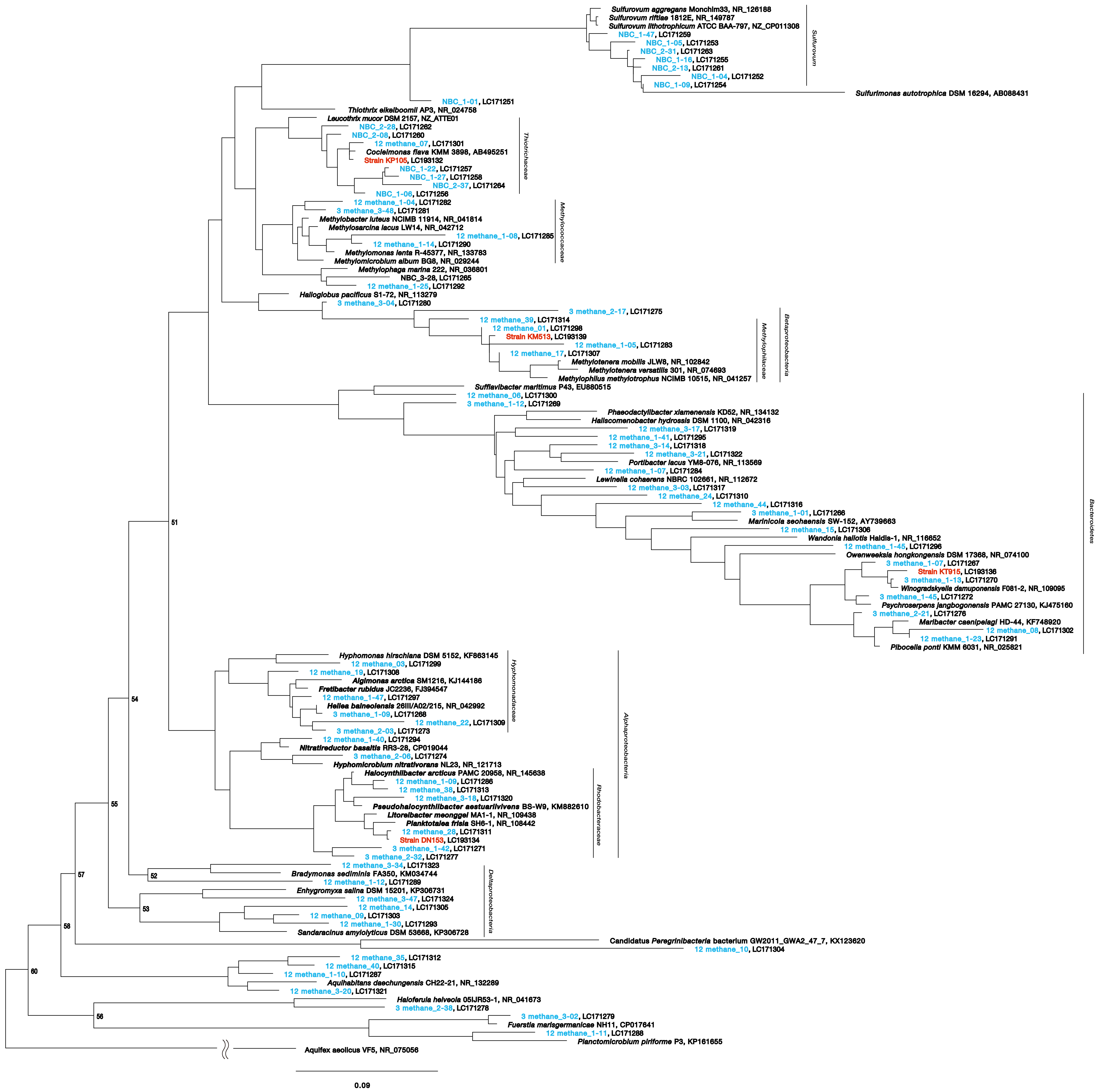

Sulfurovum aggregans Monchim33, NR_126188
Sulfurovum riftiae 1812E, NR_149787
Sulfurovum lithotrophicum ATCC BAA-797, NZ_CP011308
NBC_1-47, LC171259
NBC_1-05, LC171253
NBC_2-31, LC171263
NBC_1-16, LC171255
NBC_2-13, LC171261
NBC_1-04, LC171252
NBC_1-09, LC171254
Sulfurovum
Sulfurimonas autotrophica DSM 16294, AB088431
NBC_1-01, LC171251
Thiothrix eikelboomii AP3, NR_024758
Leucothrix mucor DSM 2157, NZ_ATTE01
NBC_2-28, LC171262
NBC_2-08, LC171260
12 methane_07, LC171301
Cocleimonas flava KMM 3898, AB495251
Strain KP105, LC193132
NBC_1-22, LC171257
NBC_1-27, LC171258
NBC_2-37, LC171264
NBC_1-06, LC171256
Thiotrichaceae
12 methane_1-04, LC171282
3 methane_3-48, LC171281
Methylobacter luteus NCIMB 11914, NR_041814
Methylosarcina lacus LW14, NR_042712
12 methane_1-08, LC171285
12 methane_1-14, LC171290
Methylomonas lenta R-45377, NR_133783
Methylomicrobium album BG8, NR_029244
Methylococcaceae
Methylophaga marina 222, NR_036801
NBC_3-28, LC171265
12 methane_1-25, LC171292
Haliglobus pacificus S1-72, NR_113279
3 methane_3-04, LC171280
3 methane_2-17, LC171275
12 methane_39, LC171314
12 methane_01, LC171298
Strain KM513, LC193139
12 methane_1-05, LC171283
12 methane_17, LC171307
Methylotenera mobilis JLW8, NR_102842
Methylotenera versatilis 301, NR_074693
Methylophilus methylotrophus NCIMB 10515, NR_041257
Methylophilaceae
Betaproteobacteria
Sufflavibacter maritimus P43, EU880515
12 methane_06, LC171300
3 methane_1-12, LC171269
Phaeodactylibacter xiamenensis KD52, NR_134132
Haliscomenobacter hydrossis DSM 1100, NR_042316
12 methane_3-17, LC171319
12 methane_1-41, LC171295
12 methane_3-14, LC171318
12 methane_3-21, LC171322
Portibacter lacus YM8-076, NR_113569
12 methane_1-07, LC171284
Lewinella cohaerens NBRC 102661, NR_112672
12 methane_3-03, LC171317
12 methane_24, LC171310
12 methane_44, LC171316
3 methane_1-01, LC171266
Marinicola seohaensis SW-152, AY739663
12 methane_15, LC171306
Wandonia haliotis Haldis-1, NR_116652
12 methane_1-45, LC171296
Owenweeksia hongkongensis DSM 17368, NR_074100
3 methane_1-07, LC171267
Strain KT915, LC193136
3 methane_1-13, LC171270
Winogradskyella damuponensis F081-2, NR_109095
3 methane_1-45, LC171272
Psychroserpens jangbogonensis PAMC 27130, KJ475160
3 methane_2-21, LC171276
Maribacter caenipelagi HD-44, KF748920
12 methane_08, LC171302
12 methane_1-23, LC171291
Pibocella ponti KMM 6031, NR_025821
Bacteroidetes
51
Hyphomonas hirschiana DSM 5152, KF863145
12 methane_03, LC171299
12 methane_19, LC171308
Algimonas arctica SM1216, KJ144186
Fretibacter rubidus JC2236, FJ394547
12 methane_1-47, LC171297
Hellea balneolensis 26III/A02/215, NR_042992
3 methane_1-09, LC171268
12 methane_22, LC171309
3 methane_2-03, LC171273
Hyphomonadaceae
12 methane_1-40, LC171294
Nitratireductor basaltis RR3-28, CP019044
3 methane_2-06, LC171274
Hyphomicrobium nitrativorans NL23, NR_121713
Halocynthiibacter arcticus PAMC 20958, NR_145638
12 methane_1-09, LC171286
12 methane_38, LC171313
12 methane_3-18, LC171320
Pseudohalocynthiibacter aestuariivivens BS-W9, KM882610
Litoreibacter meonggei MA1-1, NR_109438
Planktotalea frisia SH6-1, NR_108442
12 methane_28, LC171311
Strain DN153, LC193134
3 methane_1-42, LC171271
3 methane_2-32, LC171277
Rhodobacteraceae
Alphaproteobacteria
54
12 methane_3-34, LC171323
52
Bradymonas sediminis FA350, KM034744
12 methane_1-12, LC171289
Enhygromyxa salina DSM 15201, KP306731
12 methane_3-47, LC171324
12 methane_14, LC171305
12 methane_09, LC171303
12 methane_1-30, LC171293
Sandaracinus amylolyticus DSM 53668, KP306728
Deltaproteobacteria
53
55
57
58
Candidatus Peregrinibacteria bacterium GW2011_GWA2_47_7, KX123620
12 methane_10, LC171304
12 methane_35, LC171312
12 methane_40, LC171315
12 methane_1-10, LC171287
Aquihabitans daechungensis CH22-21, NR_132289
12 methane_3-20, LC171321
60
Haloferula helveola 05IJR53-1, NR_041673
3 methane_2-38, LC171278
56
3 methane_3-02, LC171279
Fuerstia marisgermanicae NH11, CP017641
12 methane_1-11, LC171288
Planctomicrobium piriforme P3, KP161655
Aquifex aeolicus VF5, NR_075056
0.09

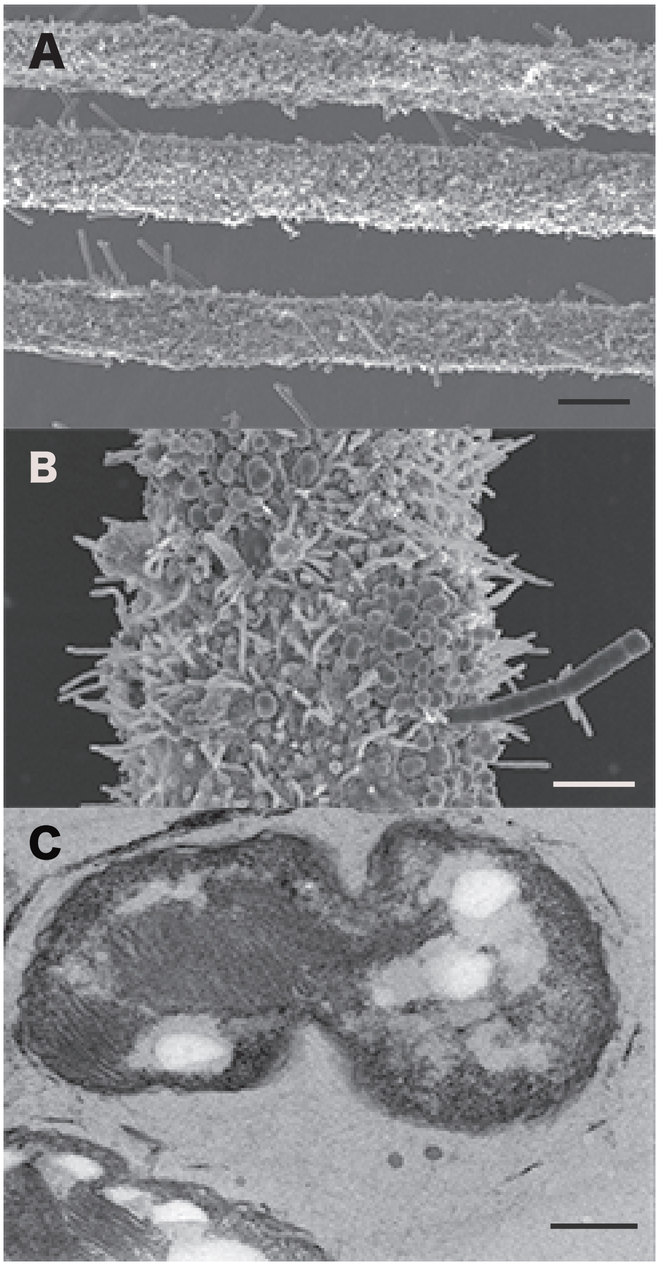
A
B
C

Table S1: Sequence similarity and compositions of bacterial phylotypes obtained from *S. crosnieri* epibiotic community.

**Group**		**Clones for the rearing periods (month)**			**Most similar sequence**		
**Phylotype ID**	**Accession No.**	**0**	**3**	**12**	**Source**	**Accession No.**	**% sim**
Epsilonproteobacteria							
(*Sulfurovum*)							
NBC_1-05	LC171253	5	0	0	Inactive chimney from East Pacific Rise	JQ287048	97
NBC_1-09	LC171254	25	0	0	*S. crosnieri* epibiotic community	AB440170	99
NBC_1-47	LC171259	7	0	0	*S. crosnieri* epibiotic community	AB981871	99
NBC_2-13	LC171261	2	0	0	*S. crosnieri* epibiotic community	AB981795	99
NBC_2-31	LC171263	2	0	0	*K. puravida* epibiotic community	JN255991	99
NBC_1-04	LC171252	3	0	0	*S. crosnieri* epibiotic community	AB981804	98
NBC_1-16	LC171255	11	0	0	*S. crosnieri* epibiotic community	AB981807	99
Gammaproteobacteria							
(*Thiotrichaceae*)							
12 methane_07	LC171301	1	7	17	*Cocleimonas flava* KMM 3898	AB495251	97
NBC_1-06	LC171256	4	0	0	*S. crosnieri* epibiotic community	EU107481	98
NBC_1-22	LC171257	5	0	0	*S. crosnieri* epibiotic community	AB476179	99
NBC_2-28	LC171262	1	0	0	*S. crosnieri* epibiotic community	AB980114	97
NBC_2-37	LC171264	1	0	0	*S. crosnieri* epibiotic community	AB981870	96
NBC_1-27	LC171258	1	0	0	*S. crosnieri* epibiotic community	AB981808	99
NBC_2-08	LC171260	3	0	0	*S. crosnieri* epibiotic community	AB981826	99
(*Methylococcaceae*)							
12 methane_1-04	LC171282	0	0	3	*S. crosnieri* epibiotic community	AB980112	99
12 methane_1-14	LC171290	7	21	4	*S. crosnieri* epibiotic community	AB476240	97
12 methane_1-08	LC171285	0	3	3	*S. crosnieri* epibiotic community	AB981805	96
3 methane_3-48	LC171281	0	1	0	Carbonates from methane seep	KM356316	99

(*Piscirickettsiaceae*)							
12 methane_1-25	LC171292	0	6	3	Borehole on Costa Rica Rift flank	GQ903367	98
NBC_3-28	LC171265	1	0	0	*S. crosnieri* epibiotic community	AB981852	98
(*Halieaceae*)							
3 methane_3-04	LC171280	0	2	0	Gut of *S. crosnieri*	AB980096	95
(Unclassified)							
NBC_1-01	LC171251	19	0	0	*S. crosnieri* epibiotic community	EU107485	97
Alphaproteobacteria							
(*Hyphomonadaceae*)							
12 methane_19	LC171308	0	0	1	*Hyphomonadaceae* bacterium JC2234	FJ394548	95
3 methane_1-09	LC171268	0	2	0	*Hellea balneolensis*	NR_042992	99
12 methane_1-47	LC171297	0	0	1	Marine biofouling	GQ274221	96
3 methane_2-03	LC171273	0	2	0	Marine biofouling	GQ274254	96
12 methane_22	LC171309	0	0	1	Hydrothermal vent biofilm	KP896648	99
12 methane_03	LC171299	0	3	2	Inactive chimney from East Pacific Rise	JQ287328	95
(*Hyphomicrobiaceae*)							
3 methane_2-06	LC171274	0	1	0	Seafloor lavas from the East Pacific Rise	EU491743	99
(*Phyllobacteriaceae*)							
12 methane_1-40	LC171294	0	0	1	Inactive chimney from East Pacific Rise	JQ287050	99
(*Rhodobacteraceae*)							
12 methane_28	LC171311	0	3	4	*Planktotalea* sp. KHS07	KP172216	99
12 methane_38	LC171313	0	0	1	*S. crosnieri* epibiotic community	AB981848	98
12 methane_1-09	LC171286	0	2	3	*S. crosnieri* epibiotic community	AB981848	98
12 methane_3-18	LC171320	0	0	1	Sediment in arctic fjord	KJ566300	98
3 methane_1-42	LC171271	0	3	0	*S. crosnieri* epibiotic community	AB981886	99
3 methane_2-32	LC171277	0	1	0	Mud volcano sediment in Mediterranean Sea	KF440308	99
Betaproteobacteria							
(*Methylophilaceae*)							
12 methane_01	LC171298	0	29	29	Gut of ascidian *Ciona intestinalis*	KF799039	99
12 methane_1-05	LC171283	0	0	2	Gut of ascidian *Ciona intestinalis*	KF799039	92

12 methane_39	LC171314	0	0	1	Bacterial community in acidification experiment	JN976619	95
12 methane_17	LC171307	0	0	1	Bacterial community co-existing with Pseudo-nitzschia	HM140666	99
(Unclassified)							
3 methane_2-17	LC171275	0	1	0	Marine sponge	EU236422	89
Deltaproteobacteria							
(*Bdellovibrionaceae*)							
12 methane_1-12	LC171289	0	0	3	Microbial mat in hypersaline pond	JN430263	93
(*Bradymonadales*)							
12 methane_3-34	LC171323	0	0	1	River sediment	KM823720	90
(*Myxococcales*)							
12 methane_3-47	LC171324	0	0	1	Marine tidal mat	AY193162	95
12 methane_14	LC171305	0	0	1	Coastal sediment	GU230393	91
12 methane_09	LC171303	0	0	1	Arctic pack ice	AF468246	99
12 methane_1-30	LC171293	0	0	1	Gut of *S. crosnieri*	AB980107	94
Bacteroidetes							
(*Cyclobacteriaceae*)							
3 methane_1-01	LC171266	0	1	0	Inactive chimney from East Pacific Rise	JQ287020	99
(*Flavobacteriaceae*)							
12 methane_1-23	LC171291	0	5	10	*Maribacter* sp. BSw21903	JQ069963	99
3 methane_2-21	LC171276	0	1	0	*Pibocella ponti*	NR_025821	97
3 methane_1-45	LC171272	0	2	0	*Psychroserpens* sp. PAMC 27104	KJ475158	99
12 methane_06	LC171300	0	0	3	methane seep sediments	FM179907	90
3 methane_1-07	LC171267	0	3	0	*Arcticiflavibacter luteus*	KF711856	99
3 methane_1-13	LC171270	0	4	0	*Winogradskyella damuponensis.*	NR_109095	98
12 methane_08	LC171302	0	0	1	Seafloor basalt	KT748595	97
(*Schleiferiaceae*)							
12 methane_1-45	LC171296	0	0	1	Macroalgal surface	GU451471	96
(*Saprospiraceae*)							
12 methane_3-14	LC171318	0	0	1	Abdominal tuft of *Pestarella tyrrhena*	DQ890438	97
12 methane_1-41	LC171295	0	0	3	Microbial mat from shallow hydrothermal vent	HQ153931	94

12 methane_24	LC171310	0	0	1	Marine biofilms	JF272143	92
12 methane_3-17	LC171319	0	1	1	Macroalgal surface	GU451380	95
12 methane_3-21	LC171322	0	0	1	Antarctic sea water	GU235293	99
12 methane_1-07	LC171284	0	0	10	Macroalgal surface	GU451530	99
12 methane_3-03	LC171317	0	0	1	Seawater from marineland for shrimp and crab	JN119168	96
3 methane_1-12	LC171269	0	6	0	Atlantic Ocean	FQ032814	94
(*Sphingobacteriales*)							
12 methane_15	LC171306	0	1	1	Atlantic Ocean	FQ032816	99
12 methane_44	LC171316	0	0	1	Coastal seawater	GQ347704	97
Planctomycetales							
12 methane_1-11	LC171288	0	0	1	*S. crosnieri* epibiotic community	AB981888	96
3 methane_3-02	LC171279	0	2	0	Microbialites from Alchichica lake	JN825603	96
Verrucomicrobiales							
3 methane_2-38	LC171278	0	1	0	*Verrucomicrobia* bacterium PAORIC-15	KP030836	99
Gracilibacteria							
12 methane_10	LC171304	0	0	1	Volcanic seamount	FJ497648	94
Acidimicrobiales							
12 methane_1-10	LC171287	0	1	2	Marine bulk water	JX016574	99
12 methane_3-20	LC171321	0	1	1	Marine subsurface sediment	KR825135	99
12 methane_35	LC171312	0	0	1	Marine sediment	FJ716840	97
12 methane_40	LC171315	0	0	1	Surface water from Bering Sea	GQ472798	98
**Total**		98	116	128			
